# Measures of Thyroid Function among Belarusian Children and Adolescents Exposed to Iodine-131 from the Accident at the Chernobyl Nuclear Plant

**DOI:** 10.1289/ehp.1205783

**Published:** 2013-05-07

**Authors:** Evgenia Ostroumova, Alexander Rozhko, Maureen Hatch, Kyoji Furukawa, Olga Polyanskaya, Robert J. McConnell, Eldar Nadyrov, Sergey Petrenko, George Romanov, Vasilina Yauseyenka, Vladimir Drozdovitch, Viktor Minenko, Alexander Prokopovich, Irina Savasteeva, Lydia B. Zablotska, Kiyohiko Mabuchi, Alina V. Brenner

**Affiliations:** 1 Division of Cancer Epidemiology and Genetics, National Cancer Institute, National Institutes of Health, Department of Health and Human Services, Bethesda, Maryland, USA; 2 The Republican Research Center for Radiation Medicine and Human Ecology, Gomel, Belarus; 3 Department of Statistics, Radiation Effects Research Foundation, Hiroshima, Japan; 4 The Thyroid Center, Columbia University, New York, New York, USA; 5 Department of Anthropoecology and Epidemiology, International Sakharov Environmental University, Minsk, Belarus; 6 Belarusian Medical Academy of Post-Graduate Education, Minsk, Belarus; 7 Department of Epidemiology and Biostatistics, University of California, San Francisco, San Francisco, California, USA

**Keywords:** antithyroid antibodies, autoimmune thyroiditis, Chernobyl, Chornobyl, dose response, hyperthyroidism, hypothyroidism, radioiodine, thyroid gland

## Abstract

Background: Thyroid dysfunction after exposure to low or moderate doses of radioactive iodine-131 (^131^I) at a young age is a public health concern. However, quantitative data are sparse concerning ^131^I-related risk of these common diseases.

Objective: Our goal was to assess the prevalence of thyroid dysfunction in association with ^131^I exposure during childhood (≤ 18 years) due to fallout from the Chernobyl accident.

Methods: We conducted a cross-sectional analysis of hypothyroidism, hyperthyroidism, autoimmune thyroiditis (AIT), serum concentrations of thyroid-stimulating hormone (TSH), and autoantibodies to thyroperoxidase (ATPO) in relation to measurement-based ^131^I dose estimates in a Belarusian cohort of 10,827 individuals screened for various thyroid diseases.

Results: Mean age at exposure (± SD) was 8.2 ± 5.0 years. Mean (median) estimated ^131^I thyroid dose was 0.54 (0.23) Gy (range, 0.001–26.6 Gy). We found significant positive associations of ^131^I dose with hypothyroidism (mainly subclinical and antibody-negative) and serum TSH concentration. The excess odds ratio per 1 Gy for hypothyroidism was 0.34 (95% CI: 0.15, 0.62) and varied significantly by age at exposure and at examination, presence of goiter, and urban/rural residency. We found no evidence of positive associations with antibody-positive hypothyroidism, hyperthyroidism, AIT, or elevated ATPO.

Conclusions: The association between ^131^I dose and hypothyroidism in the Belarusian cohort is consistent with that previously reported for a Ukrainian cohort and strengthens evidence of the effect of environmental ^131^I exposure during childhood on hypothyroidism, but not other thyroid outcomes.

The most severe accident in the history of nuclear industry occurred on 26 April 1986, at the Chernobyl nuclear power plant located in Ukraine, about 10 km south of the border with Belarus. The radioactive fallout contained short-lived, mainly radioiodines, and long-lived radionuclides such as cesium-137 (^137^Cs). Overall, 1,760 petabecquerel (PBq = 10^15^ Bq) of iodine-131 (^131^I) and 85 PBq of ^137^Cs were released into the environment [United Nations Scientific Committee on the Effects of Atomic Radiation (UNSCEAR) 2010].

Persons ≤ 18 years of age at the time of the accident received substantial thyroid doses of ^131^I due to consumption of contaminated milk and dairy products and relatively small size of the thyroid gland. An association has been established between ^131^I exposure during childhood and thyroid cancer risk in Ukraine, Belarus, and the Bryansk region of the Russian Federation (Brenner et al. 2011; Cardis et al. 2005; Kopecky et al. 2006; Tronko et al. 2006b; Zablotska et al. 2011). Two decades after the accident there is no evidence of a decline in radiation-related risk of thyroid cancer (Brenner et al. 2011).

Post-Chernobyl consequences of childhood ^131^I exposure on thyroid function are less clear, but diseases related to thyroid function (e.g., hypothyroidism, hyperthyroidism) are more common than thyroid cancer and could result in substantial morbidity among those exposed. Some earlier studies reported an increase in thyroid-stimulating hormone (TSH) levels and the prevalence of juvenile hypothyroidism among children exposed to ^131^I following Chernobyl (Goldsmith et al. 1999; Quastel et al. 1997; Vykhovanets et al. 1997), but other studies did not (Agate et al. 2008; Kasatkina et al. 1997; Pacini et al. 1998; Vermiglio et al. 1999). An increased prevalence of thyroid autoimmunity has been reported (Kasatkina et al. 1997; Pacini et al. 1998; Vermiglio et al. 1999), although it seemed to be transient with no long-term effect on thyroid function (Agate et al. 2008). Most studies published to date, except for the cohort study in Ukraine described below, were limited in size or lacked individual dose estimates.

In a screening study among 12,000 subjects in Ukraine with doses estimated from individual measurements of thyroid radioactivity, significant associations were found between ^131^I thyroid dose (mean dose of 0.79 Gy) and prevalence of subclinical hypothyroidism (Ostroumova et al. 2009) and antibodies to thyroperoxidase (ATPO) (Tronko et al. 2006a), but not autoimmune thyroiditis (AIT) (Tronko et al. 2006a) or hyperthyroidism (Hatch et al. 2010).

To extend findings from the Ukrainian cohort, we evaluated functional thyroid outcomes in relation to individual ^131^I thyroid doses in a comparable cohort of exposed children and adolescents from Belarus who were screened for thyroid cancer and other thyroid diseases. The methods used to estimate thyroid doses and to screen for thyroid diseases in Belarus and Ukraine were similar (Stezhko et al. 2004).

## Methods

*Study population*. The Belarus cohort consists of individuals who were ≤ 18 years of age on 26 April 1986 and had their thyroid radioactivity measured within 2 months of the accident. Study design and methods have been described in detail by Stezhko et al. (2004). The study protocol called for a standardized, in-depth thyroid screening examination every 2 years. In total, 38,543 eligible individuals were identified. Of these, 16,213 were traced through address bureaus, military registration offices, departments of education and public health, and medical establishments, and sent invitation letters explaining the study. The present analysis is based on the data collected from the 11,970 traceable cohort members who attended the first screening examination conducted between 1996 and 2003. Of 11,970 individuals examined in the first screening cycle, we sequentially excluded from the analytic cohort 1,143 participants because of incorrect identification (*n* = 20); noneligible age (*n* = 114); inadequate dose estimates (*n* = 104); self-reported history of any thyroid disease before screening examination including, for example, nodular or diffuse goiter, thyroiditis, hypo- and hyperthyroidism (*n* = 542), or benign thyroid surgery (*n* = 58) or thyroid hormone intake (*n* = 168); lack of TSH measurements (*n* = 59) or TSH measured using AxSYM method (Abbott Laboratories. Abbott Park, IL, USA) (*n* = 12); or lack of thyroid volume measurement (*n* = 66). The final sample for analysis was 10,827.

The study was reviewed and approved by the institutional review boards of the participating organizations in Belarus and the United States, and all study participants or their guardians (for subjects who were ≤ 16 years of age at screening) signed informed consent.

*Screening procedure*. Individuals were screened in one of the two fixed centers in Minsk or Gomel or by mobile teams at local medical facilities. The screening procedure included thyroid palpation and ultrasonographic examination by an ultrasonographer, independent thyroid palpation by an endocrinologist, collection of sociodemographic information and medical history data, collection of blood sample and spot urine sample, and dosimetry interview. In the case of thyroid nodule or focal lesion with the largest diameter ≥ 10 mm detected by either palpation or ultrasonography, thyroid nodule 5–10 mm with ultrasound evidence of malignancy (indistinct borders, extension through thyroid capsule, heterogeneous or hypoechoic density, stippled calcification, increasing size during follow-up, or abnormal lymph nodes), or diffusely abnormal thyroid structure accompanied by unexplained cervical lymphadenopathy, the study subject was referred for fine needle aspiration biopsy (Stezhko et al. 2004).

*Laboratory methods*. For most cohort members, TSH, ATPO, free thyroxine (FT_4_), and autoantibodies to thyroglobulin (ATG) were measured in serum samples with LUMitest immunochemiluminescence assays (BRAHMS Diagnostica GmbH, Henningsdorf, Germany) using a Berthold 953 luminometer (Berthold Technologies, GmbH & Co. KG, Bad Wildbad, Pforzheim, Germany). TSH, ATPO, and ATG concentrations were measured in each study subject with a sufficient serum sample, while FT_4_ was measured only in those with TSH levels outside the reference range. For a portion of the cohort, TSH (*n* = 3,501) and FT_4_ (*n* = 311) were measured with IMx immunochemiluminescence assays (Abbott Laboratories) using a Berthold 953 luminometer. All assays were conducted according to the manufacturer’s instruction.

Urinary iodine concentrations (micrograms per liter) were measured photometrically using the Sandell–Kolthoff reaction as modified by Dunn et al. (1993). The analytical sensitivity of the assay was 10 µg/L.

*Diagnostic criteria*. After evaluating the range of TSH values in a reference sample from our cohort, we set reference limits for serum TSH concentration between 0.3 and 4.0 mIU/L. We defined hypothyroidism as a serum TSH concentration > 4 mIU/L, the upper limit of the reference range. Participants with serum TSH concentration > 4 mIU/L and FT_4_ < 10 pmol/L were considered to be cases of overt hypothyroidism. Hyperthyroidism was defined as serum TSH concentration < 0.3 mIU/L, the lower limit of the reference range, and participants with FT_4_ > 25 pmol/L were considered to be cases of overt hyperthyroidism. Elevated levels of ATPO (ATPO-positivity) and ATG (ATG-positivity) were defined as ATPO > 60 U/mL and ATG > 60 U/mL, respectively, consistent with BRAHMS recommendation. Participants with serum TSH concentration > 4 mIU/L and ATPO > 60 U/mL were considered to be cases of antibody-positive hypothyroidism. Autoimmune thyroiditis (AIT) was defined based on a combination of laboratory (elevated TSH, ATPO, or ATG concentrations), ultrasound (hypoechoic gland with heterogeneous/granular structure) and palpatory (firm gland) findings, as described elsewhere (Tronko et al. 2006a).

*Dosimetry*. ^131^I dose reconstruction methods have been described in detail by Drozdovitch et al. (2013). In brief, individual ^131^I thyroid doses were estimated based on direct thyroid radioactivity measurements performed at least once for each cohort member in May–June 1986 (Gavrilin et al. 1999); personal interview information on residences, dietary habits, and administration of potassium iodide promptly after the accident; and a radioecological model that was used to estimate temporal variation of ^131^I in the thyroid gland. For subjects < 10 years of age at the time of the accident, the dosimetry interview was conducted with the subject’s mother or other close relative.

Intake of ^131^I, mainly through milk consumption, accounted for about 95% of the estimated thyroid dose. Other pathways of exposure, intakes of short-lived radioiodines, external irradiation from radionuclides deposited on the ground, and intakes of long-lived ^134^Cs and ^137^Cs, were minor contributors to the estimated ^131^I dose (Bouville et al. 2007).

*Statistical methods*. We estimated odds ratios (ORs) and computed corresponding 95% CIs based on logistic regression analysis using the GMBO module of Epicure statistical software (Preston et al. 1993). We assumed that the odds of each studied thyroid outcome, γ(*x, d*), depended on a vector of covariates *x* that described the background (in the absence of ^131^I exposure) prevalence, and the estimated ^131^I thyroid dose *d*. For modeling prevalence of a specific thyroid outcome (e.g., hypothyroidism), we did not exclude participants with other thyroid outcomes from the analysis.

The background adjustment factors were outcome-specific and included sex, age at examination (10–14, 15–19, 20–24, ≥ 25 years), oblast (an administrative subdivision similar to a state or province) of residency at examination, rural or urban residency at examination, self-reported current cigarette smoking, self-reported current vitamin consumption, self-reported history of any thyroid disease in parents or siblings, year and season of examination, level of urinary iodine (< 20, 20–49, 50–99, ≥ 100 µg/L, or unknown), presence of diffuse goiter based on thyroid palpation, ATPO and ATG concentrations (≤ 60, > 60 U/mL). For each studied outcome, we retained in the final model those background factors whose inclusion into the model significantly improved the model fit, based on the likelihood ratio test comparing models with and without the covariate included at the 0.05 alpha level. We also retained those factors previously associated with the outcome of interest in non-irradiated populations.

Under a multiplicative OR model, γ can be written as a product of background prevalence odds of a specific thyroid outcome, denoted as γ_0_(*x*), and a dose–response function, h(*d*). We fitted a simple linear dose–response model, γ(*x,d*) *=* γ_0_(*x*) *×* (1 + β*d*), where β is the excess odds ratio (EOR), the parameter that measures the increase in EOR per unit increase in dose (EOR/Gy). We evaluated departure from linearity by fitting a linear-quadratic γ(*x,d*) *=* γ_0_(*x*) *×* (1 + β*d +* θ*d^2^*) dose–response model. We also evaluated a categorical dose–response model using these categories—< 0.1–0.249, 0.25–0.49, 0.5–0.99, 1.0–2.49, 2.50–4.99, 5.00–9.99, ≥ 10.0 Gy—to assure a reasonably proportional increment in ^131^I dose. In addition to analysis of hypothyroidism on dichotomous scale (TSH ≤ 4.0 vs. TSH > 4.0 mIU/L), we analyzed continuous TSH concentrations using generalized linear regression models, where the mean of ln(TSH + 1) was described as a linear function of the same background adjustment factors used in the logistic model and ^131^I dose. We fitted statistical models over the entire dose range and performed sensitivity analyses excluding subjects with high ^131^I doses of ≥ 10 Gy and ≥ 5 Gy. We tested for dose–response trends by modeling exposure both as a continuous variable and as an ordinal categorical variable coded using integer values (0–7).

To evaluate factors interacting with dose, we allowed a linear dose–response trend β to vary within *J* categories of different factors such as sex, age at exposure and examination, current smoking status, urban versus rural residency, oblast of residence at first screening examination, family history of thyroid disease, ATPO level, presence of goiter, and urinary iodine levels. We compared two nested models with and without an interaction term between dose and factor under investigation using likelihood ratio test with *J* – 1 degrees of freedom (df). A significant *p*-value indicated that the association between radiation and the prevalence of the outcome was not homogeneous across levels of the factor of interest.

We estimated attributable risk at a given dose level as the ratio of excess cases to observed cases expressed as percentage. Excess cases were estimated as the difference between the numbers of observed and expected (in the absence of radiation exposure) cases.

We estimated the statistical significance of model parameters, test of trend, and comparison in goodness of fit between models using likelihood ratio chi-square tests with df equal to the difference in number of parameters between the models being compared. All tests were two-sided, and we considered *p* < 0.05 to be statistically significant.

## Results

*Characteristics of study participants and prevalence of functional thyroid outcomes*. The main characteristics of the study cohort (*n* = 10,827) are summarized in Table 1. Women represented 50% of the cohort. Most study participants were exposed at ages < 10 years (62%) and were ≥ 20 years of age at the time of the first screening examination (58%). The first screening cycle took place from 1996 through 2003, and 86% of the study subjects were screened in 1996–2000. At the time of screening 60% of the cohort resided in Gomel oblast. The mean (median) ^131^I thyroid dose was 0.54 (0.23) Gy, ranging from 0.001 to 26.6 Gy.

**Table 1:** Main characteristics of the study cohort.

Characteristic	*n* (%)
Sex
Male	5,400 (49.9)
Female	5,427 (50.1)
Age at exposure (years)
≤4	3,462 (32.0)
5–9	3,248 (30.0)
10–14	2,853 (26.3)
15–18	1,264 (11.7)
Mean±SD	8.24±4.95
Age at examination (years)
10–14	1,257 (11.6)
15–19	3,263 (30.1)
20–24	3,054 (28.2)
≥25	3,253 (30.0)
Mean±SD	21.65±5.28
Year of examination
1996–1998	2,967 (27.4)
1999	2,268 (21.0)
2000	4,071 (37.6)
2001–2003	1,521 (14.0)
Place of residency at examination
Gomel oblast	6,511 (60.1)
Minsk oblast and Minsk City	3,113 (28.8)
Mogilev oblast	779 (7.2)
Other oblasts	424 (3.9)
Thyroid dose (Gy)
0.001–0.049	1,960 (18.1)
0.05–0.099	1,339 (12.4)
0.10–0.249	2,398 (22.1)
0.25–0.49	2,026 (18.7)
0.50–0.99	1,589 (14.7)
1.00–2.49	1,138 (10.5)
2.50–4.99	280 (2.6)
5.00–26.64	97 (0.9)
Mean (median)	0.54 (0.23)
Total	10,827 (100.0)

The prevalence of functional thyroid outcomes in the study cohort was as follows: 2.95% for hypothyroidism (*n* = 319 including 18 cases of overt hypothyroidism); 1.26% for hyperthyroidism (*n* = 137 including 13 cases of overt hyperthyroidism); 5.74% for elevated ATPO (*n* = 622); and 0.80% for AIT (*n* = 87).

**Figure 1:**
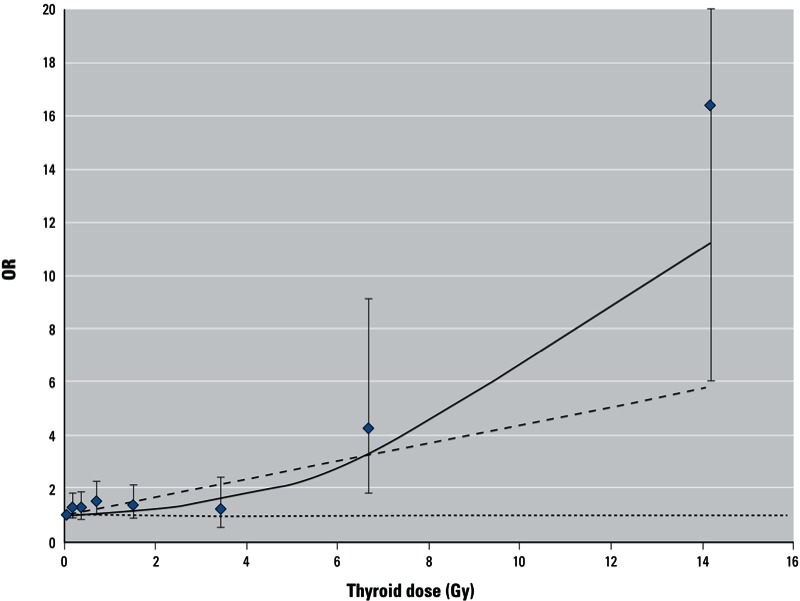
Dose–response association between prevalence of hypothyroidism (serum TSH > 4 mIU/L) and ^131^I thyroid dose estimates in a cohort study of thyroid cancer and other thyroid diseases after the Chernobyl accident in Belarus, 1996–2003. Dose–response line was adjusted to pass through the lowest ^131^I dose category. Data points are ^131^I dose category-specific ORs with 95% CIs (whiskers). Curves represent fitted ORs based on linear (dotted line) and linear-quadratic (solid line) EOR model.

*Associations between ^131^I dose and thyroid outcomes*. Estimated ORs by categories of ^131^I thyroid dose and EORs/Gy based on a simple linear model adjusted for background factors are shown in Table 2, and associations between background factors and each outcome (excluding antibody-positive hypothyroidism) and between age group and each outcome according to sex are shown in Supplemental Material, Tables S1 and S2, respectively (http://dx.doi.org/10.1289/ehp.1205783). We found a nonmonotonic but significant positive trend with increasing ^131^I dose for hypothyroidism (*p* < 0.001) and a nonmonotonic nonsignificant positive trend for AIT (*p* = 0.07), but did not find evidence of positive trends for the other outcomes. Although the EOR/Gy for elevated ATPO levels was –0.07 (95% CI: –0.16, –0.005), this finding was driven by a small number of cases exposed to high doses of ^131^I (*n* = 2), and the overall trend was nonsignificant (*p* = 0.30). The estimated EOR/Gy for hypothyroidism was 0.34 (95% CI: 0.15, 0.62) based on a linear dose–response model. However, over the entire dose range the linear-quadratic model fit the data for hypothyroidism significantly better than a simple linear model (*p* = 0.02 for linear-quadratic vs. linear model comparison, Figure 1). When individuals with ^131^I doses ≥ 10 Gy were excluded (23 subjects including 9 cases of hypothyroidism), there was no significant difference in fit between the two models (*p* = 0.69 for linear-quadratic vs. linear model comparison). The estimate of the EOR/Gy at a dose range < 10 Gy was 0.21 (95% CI: 0.04, 0.47), somewhat lower than the linear estimate of 0.34 based on the entire dose range, yet statistically significant. The estimate of the EOR/Gy at a dose range < 5 Gy was 0.11 (95% CI: –0.07, 0.40). Based on a linear model, 36 of 319 hypothyroidism cases (11.3%) in the study could be attributed to ^131^I thyroid exposure.

**Table 2:** Association between prevalent functional thyroid outcomes and ^131^I thyroid dose estimates.

Outcome	Thyroid dose (Gy)								EOR/Gy^*b*^ (95% CI) trend *p*-value
	<0.1 (0.04)^*a*^	0.1–0.249 (0.2)	0.25–0.49 (0.4)	0.50–0.99 (0.7)	1.00–2.49 (1.5)	2.50–4.99 (3.4)	5.00–9.99 (6.7)	≥10.0(14.2)	
Hypothyroidism (*n*=319)
Cases (%)^*c*^	63 (1.9)	68 (2.8)	61 (3.0)	58 (3.7)	41 (3.6)	10 (3.7)	9 (12.2)	9 (39.1)
OR (95% CI)	1.00 (referent)	1.29 (0.89, 1.86)	1.30 (0.88, 1.90)	1.54 (1.04, 2.29)	1.39 (0.89, 2.15)	1.23 (0.57, 2.43)	4.31 (1.85, 9.15)	16.46 (6.07, 42.76)	0.34 (0.15, 0.62) *p*<0.001
Antibody-positive hypothyroidism (*n*=62)
Cases (%)	17 (0.5)	17 (0.7)	14 (0.7)	9 (0.6)	5 (0.4)	0	0	0
OR (95% CI)	1.00 (referent)	1.51 (0.77, 2.95)	1.47 (0.71, 2.98)	1.41 (0.59, 3.13)	1.40 (0.44, 3.74)	—	—	—	–0.07 (–0.35, 0.61) *p*=0.78
Hyperthyroidism (*n*=137)
Cases (%)	46 (1.4)	35 (1.5)	16 (0.8)	23 (1.4)	16 (1.4)	1 (0.4)	0	0
OR (95% CI)	1.00 (referent)	0.92 (0.58, 1.45)	0.49 (0.26, 0.87)	0.97 (0.56, 1.64)	1.07 (0.56, 1.94)	0.28 (0.02, 1.35)	—	—	–0.11 (–0.28, 0.19) *p*=0.41
Elevated ATPO (*n*=622)
Cases (%)	203 (6.2)	147 (6.1)	122 (6.0)	85 (5.3)	55 (4.8)	8 (2.9)	2 (2.7)	0
OR (95% CI)	1.00 (referent)	1.03 (0.82, 1.30)	1.02 (0.80, 1.29)	0.96 (0.73, 1.26)	0.96 (0.69, 1.32)	0.61 (0.27, 1.19)	0.62 (0.10, 2.03)	—	–0.10 (–0.16, –0.015) *p*=0.30
AIT (*n*=87)
Cases (%)	20 (0.6)	21 (0.9)	23 (1.1)	14 (0.9)	6 (0.5)	1 (0.4)	2 (2.7)	0
OR (95% CI)	1.00 (referent)	1.82 (0.97, 3.45)	2.44 (1.30, 4.61)	2.23 (1.06, 4.58)	1.50 (0.53, 3.71)	1.30 (0.07, 6.71)	10.11 (1.43, 42.92)	—	0.24 (–0.08, 1.06) *p*=0.07
^***a***^Mean estimate in each thyroid dose category (Gy). ^***b***^Excess odds ratio (EOR) based on a linear dose–response model with adjustment for the following: for hypothyroidism, sex, age at examination by sex, oblast of residency, rural or urban residency, current smoking, year of examination, ATPO and ATG levels, urinary iodine excretion level, presence of goiter; for antibody-positive hypothyroidism, sex, age at examination, current smoking, urinary iodine excretion level, presence of goiter; for hyperthyroidism, sex, age at examination by sex, urban or rural residency, year of examination, ATPO level, urinary iodine excretion level, presence of goiter; for elevated ATPO, sex, age at examination by sex, urban or rural residency, current smoking, year and season of examination, presence of goiter; for AIT, sex, age at examination by sex, urban or rural residency, urinary iodine excretion level, presence of goiter. Trend *p*-values df=1. ^***c***^Percent of individuals with the respective outcome in each thyroid dose category.									

Analysis of TSH levels on a continuous scale using generalized linear models provided similar results to those based on the dichotomous definition of hypothyroidism: There was a significant increase in TSH concentration with increasing ^131^I thyroid dose over the entire dose range that was best described by the linear-quadratic function (*p* < 0.001 for linear-quadratic vs. linear model comparison) (data not shown). The estimated coefficient for an increase in TSH concentration per 1 Gy based on the linear model was 0.03 (95% CI: 0.02, 0.05). In contrast to the analysis of TSH on a dichotomous scale, continuous TSH analysis showed a significant positive dose–response with ^131^I not only in the range of thyroid doses up to 10 Gy, but also in the range of doses up to 5 Gy (*p* for linear trend = 0.004). No evidence of better fit of linear-quadratic compared to a simple linear dose–response model was found at ^131^I thyroid doses < 5 Gy (*p* for two model comparison = 0.85). The estimated coefficient for an increase in TSH concentration per 1 Gy based on the linear model in the dose range up to 5 Gy was 0.02 (95% CI: 0.01, 0.04). After hypothyroid participants were excluded (i.e., those with TSH > 4.0 mIU/L), the estimated coefficient for TSH in association with a 1-Gy increase in exposure across the entire dose range was 0.02 (95% CI: 0.01, 0.03; *p* for trend < 0.001).

Analyses of AIT and other outcomes using nonlinear dose–response models or a limited dose range did not provide evidence of significant positive associations with ^131^I.

*Modification of associations between ^131^I dose and hypothyroidism*. Estimates of the linear dose–response slope for prevalent hypothyroidism by selected characteristics are summarized in Table 3. The EOR/Gy did not vary significantly by sex (*p* = 0.60), cigarette smoking (*p* = 0.76), urinary iodine level (data not shown, *p* = 0.23), family history of thyroid disease (data not shown, *p* = 0.10), or oblast of residence at first screening examination (data not shown, *p* = 0.25). We found a significant interaction of ^131^I dose with both age at exposure (*p* = 0.04) and age at examination (*p* = 0.03), with EOR/Gy decreasing with increasing age at exposure or at examination. The highest EORs/Gy were found in individuals youngest at the time of the accident (< 5 years) or at the time of screening (< 20 years). We observed a significantly higher EOR/Gy for hypothyroidism in rural compared with urban residents (*p* = 0.02) and among subjects without diffuse goiter compared with subjects with diffuse goiter (*p* = 0.02). ATPO-negative individuals had higher radiation-related risk of hypothyroidism (EOR/Gy = 0.40; 95% CI: 0.19, 0.74) than ATPO-positive individuals (EOR/Gy = –0.19; 95% CI: –0.38, 0.42) but the difference was of borderline statistical significance (*p* = 0.06). Assessment of interactions between ^131^I dose and the above factors on a continuous TSH scale provided consistent results in terms of statistical significance and direction except for smoking (data not shown); the increase in TSH concentration per 1 Gy in nonsmokers (0.01; 95% CI: –0.01, 0.04) compared with smokers (0.04; 95% CI: 0.03, 0,06) was statistically significantly lower (*p* = 0.04).

**Table 3:** Effect modification of the EOR for hypothyroidism prevalence (serum TSH > 4 mIU/L) per Gy of estimated ^131^I thyroid dose according to selected characteristics.

Characteristic	Cases (*n*)	EOR/Gy (95% CI)^*a*^	*p*-Value^*b*^
Sex	0.60, df=1
Men	137	0.29 (0.08, 0.68)
Women	182	0.41 (0.14, 0.89)
Age at exposure (years)	0.04, df=2
0–4	158	0.53 (0.24, 1.00)
5–9	70	0.24 (–0.10, 0.88)
≥10	91	–0.02 (–0.16, 0.30)
Age at examination (years)	0.03, df=2
<15	89	0.47 (0.14, 1.22)
15–20	91	0.59 (0.21, 1.33)
≥20	139	–0.02 (–0.14, 0.27)
Smoking^*c*^	0.76, df=1
No	269	0.36 (0.15, 0.68)
Yes	49	0.27 (–0.01, 1.01)
ATPO level (U/mL)	0.06, df =1
≤60	257	0.40 (0.19, 0.74)
>60	62	–0.19 (–0.38, 0.42)
Urban/rural residency	0.02, df=1
Rural	161	0.59 (0.26, 1.20)
Urban	158	0.08 (–0.09, 0.38)
Presence of goiter^*d*^	0.02, df=1
No	245	0.50 (0.24, 0.90)
Yes	73	0.04 (–0.09, 0.32)
^***a***^Based on a simple linear dose–response model adjusted for sex, age at examination by sex, oblast of residency, urban or rural residency, current smoking, ATPO and ATG levels, examination year, urinary iodine excretion levels, presence of goiter. ^***b***^*p*-Value of maximum likelihood ratio test comparing the fit of models with and without interaction terms. ^***c***^Subjects with unknown smoking status excluded from the analysis. ^***d***^Subjects with unknown goiter status excluded from the analysis.			

## Discussion

We found a significant association between low to moderate ^131^I thyroid doses and prevalent, predominantly subclinical hypothyroidism in about 11,000 residents of Belarus exposed to radioactive fallout from the Chernobyl accident at ≤ 18 years of age. Over the entire dose range, a linear-quadratic dose–response model fit the data best, with the quadratic component attributed largely to a few individuals with doses ≥ 10 Gy. We also found an association with TSH levels on a continuous scale. The dose–response data for TSH were best described by a linear-quadratic function over the entire dose range, but a significant linear dose response was found in the range of doses up to 5 Gy, suggesting that levels of ^131^I an order of magnitude below the typical doses (≥ 50 Gy) used to treat thyroid disease (e.g., Graves’ disease) (Cooper and Biondi 2012; Eastman 2012) can induce functional changes in the thyroid gland. In a parallel study of about 12,000 Ukrainian residents exposed to Chernobyl fallout as children or adolescents (Ostroumova et al. 2009), the dose response for hypothyroidism was well described by a simple linear model. In the present study the EORs/Gy were 0.34 (95% CI: 0.15, 0.62) over the entire range of doses, and 0.11 (95% CI: –0.07, 0.40) at doses < 5 Gy—not meaningfully different from the respective estimates in the Ukrainian study (EOR/Gy of 0.10; 95% CI: 0.03, 0.21 over the entire dose range, and 0.16; 95% CI: 0.02, 0.34 at doses < 5 Gy).

Some previous studies of hypothyroidism or TSH levels among children exposed to radioiodines from Chernobyl fallout have reported higher TSH concentrations in exposed subjects compared with unexposed controls (Goldsmith et al. 1999; Quastel et al. 1997; Vykhovanets et al. 1997), though others have not (Agate et al. 2008; Pacini et al. 1998; Vermiglio et al. 1999). Almost all above-mentioned studies were based on small samples (from 53 to 804 exposed subjects) or lacked estimates of individual ^131^I thyroid doses. Findings from other populations exposed to ^131^I, such as Marshall Islanders (Morimoto et al. 1987; Takahashi et al. 1999), residents near the Hanford (Davis et al. 2004) and Mayak nuclear facilities (Mushkacheva et al. 2006), as well as those downwind of nuclear testing in Nevada (Lyon et al. 2006) have been primarily null, but samples sizes were also relatively small (~ 1,000–3,500 subjects), and doses were lower or not well quantified. The reports from the two large screening studies in Belarus and Ukraine provide stronger evidence than previous studies, given their individual dosimetry based on measurements taken within 2 months of the accident and the in-depth, standard protocol for case ascertainment.

We observed a significant variation in association between radiation and prevalence of hypothyroidism by age at exposure and age at examination. These two variables are closely correlated and it is not possible statistically to distinguish their independent associations. In either case, the differences according to age are consistent with higher radiation sensitivity of young thyroid tissue (Ron et al. 1995). In the Marshallese population, the most marked elevations of TSH were observed among subjects exposed at ≤ 6 years of age at doses to the thyroid from 390 to 2,100 rad (or 3.9 to 21 Gy) (Larsen et al. 1982).

In addition to variation by age, the association between radiation and prevalence of hypothyroidism also varied according to rural or urban residence and diagnosis of goiter. It is unclear what factors might be responsible for stronger associations among rural compared with urban residents and in nongoitrous compared with goitrous individuals, but differences in the past intake of dietary iodine could be important. We did not find significant variation of radiation–hypothyroidism association according to urinary iodine excretion levels, but these reflect current levels of iodine intake and are subject to high within-individual variability. Because individuals were screened over a relatively long period, we adjusted all our ^131^I risk models, except for AIT (where no significant association with calendar time was found), for possible effect of calendar time. We did not find significant differences in associations between radiation and prevalence of any of the outcomes by year of examination (data not shown).

The present study provided some evidence of a higher radiation-related risk of hypothyroidism among ATPO-negative (ATPO ≤ 60 U/mL) compared with ATPO-positive individuals (ATPO > 60 U/mL) (*p* for homogeneity = 0.06). Although in nonexposed populations autoimmune thyroid diseases or elevated thyroid antibody levels have been associated with higher risk of progression to overt hypothyroidism (Col et al. 2004; Kaplowitz 2010; Vanderpump 2011), the Ukrainian study also found a stronger dose–response relationship between hypothyroidism and ^131^I thyroid dose among ATPO-negative compared with ATPO-positive individuals (*p* for homogeneity < 0.001) (Ostroumova et al. 2009). Hence, the role of thyroid antibodies in radiation-related hypothyroidism after exposure at low to moderate ^131^I doses is not entirely clear. A transient autoimmune reaction without triggering autoimmune disease and with no effect on thyroid function has been reported in 283 exposed individuals in Belarus 13–15 years after exposure in adolescence to ^131^I from Chernobyl fallout (Agate et al. 2008). In atomic bomb survivors exposed to acute gamma radiation (Imaizumi et al. 2006; Morimoto et al. 1987; Nagataki et al. 1994), a convex dose–response association for antibody-positive hypothyroidism was shown (Nagataki et al. 1994), although not confirmed in a more recent and larger study (Imaizumi et al. 2006). More prospective data are required to improve our understanding of the complex relationship between ^131^I exposure, TSH concentration or hypothyroidism, and thyroid autoimmunity.

We found little to no evidence of positive associations between radiation and hyperthyroidism, or AIT. Similarly, no evidence of a positive association between ^131^I dose and hyperthyroidism or AIT was found in the Ukrainian cohort (Hatch et al. 2010; Tronko et al. 2006a). However, lack of a positive association between ^131^I and prevalence of ATPO in Belarus contrasts with significant positive nonlinear association with ^131^I observed in Ukraine (Tronko et al. 2006a). To better understand these differences and to evaluate the temporal trend in ATPO levels, further follow-up and analyses of prospective data in both cohorts would be necessary.

In our cohort screened at ages ranging from 11 to 33 years (mean age at examination, 22 years), hypothyroidism (94% subclinical) was diagnosed in 319 subjects (about 3% of the total). We estimated that 36 cases (11.3%) could be attributed to ^131^I exposure. The dose–response data for continuous TSH levels suggest a significant positive association between TSH and thyroid ^131^I dose even when participants classified as hypothyroid (based on TSH > 4.0 mIU/L, the upper limit of the TSH reference range) were excluded.

There is controversy in the literature about the appropriate upper TSH reference limit (Cooper and Biondi 2012; Laurberg et al. 2011; Surks et al. 2004). Calls have been made to lower the current upper TSH reference limit from 4.0 mIU/L to 2.5 mIU/L. It has been shown that TSH > 2.5 mIU/L with or without the presence of antithyroid antibodies is a predictor of long-term risk of clinical hypothyroidism (Li et al. 2008; Vanderpump et al. 1995; Walsh et al. 2010).

The clinical consequences of moderately elevated TSH levels are the subject of continuing discussion in the context of risks versus benefits related to treatment of subclinical hypothyroidism (Col et al. 2004; Cooper and Biondi 2012; Kaplowitz 2010; Khandelwal and Tandon 2012; Surks et al. 2004). It has been reported that 2–5% of patients with subclinical hypothyroidism, if untreated, progress to overt hypothyroidism, with the rate of progression being proportional to the baseline serum TSH concentration (Surks et al. 2004). There is also a concern regarding the association between subclinical hypothyroidism and cardiac dysfunction (Surks et al. 2004) as well as the course and outcomes of pregnancy (Cooper and Biondi 2012; Eastman 2012).

Although our study had significant strengths, there were some limitations. Among the 16,213 eligible and traced subjects, 11,970 (73.8%) underwent the first screening examination. However, self-selection bias related to dose seems unlikely because study participants and study personnel were unaware of subjects’ exposure level. We do not believe that exclusion from the study of 542 individuals with self-reported thyroid diseases diagnosed before the first screening examination biased the dose–response estimates because the EORs/Gy including and excluding these individuals were not meaningfully different for any outcome (data not shown). For example, for hypothyroidism the EORs/Gy were 0.50 (95% CI: 0.45, 0.83) and 0.34 (95% CI: 0.15, 0.62), respectively. Because diagnostic criteria and laboratory assays used to diagnose thyroid diseases preceding screening examination could vary across medical institutions and differ from our outcome definitions, we considered it appropriate to exclude the 542 subjects from the analysis. Although we used two assays to measure TSH levels, there were no differences between the groups measured by IMx and LUMI assay in overall TSH distribution or in the assays reference limits established based on evaluation of the reference sample from our cohort (data not shown). Moreover, we found no difference in the estimates of EOR/Gy for hypothyroidism in the groups measured by different TSH assays (data not shown).

At this stage we did not take into account the impact of uncertainties in dose estimates because work on assessment of thyroid dose uncertainties in the cohort is ongoing. Typically, radiation doses are estimated with a combination of Berkson and classical type measurement errors (Li et al. 2007; Mallick et al. 2002). Ignoring those errors in individual dose reconstruction results in underestimation of both the point estimate of radiation risk per Gy (as a continuous variable) and the upper limit of 95% CI (Li et al. 2007). Thus, the true association between radiation and hypothyroidism may be stronger than the estimate we report here.

## Conclusions

We found significant positive associations between estimated ^131^I thyroid dose from the Chernobyl accident and prevalence of hypothyroidism (defined as serum TSH > 4.0 mIU/L) or serum TSH concentration among subjects exposed at ≤ 18 years of age. The excess OR per 1 Gy for hypothyroidism was 0.34 (95% CI: 0.15, 0.62). Associations between radiation and prevalent hypothyroidism were stronger among younger individuals and those with ATPO levels ≤ 60 U/mL. No significant positive associations were observed between radioiodine exposure and antibody-positive hypothyroidism, thyroid autoimmunity, or hyperthyroidism. Further analysis incorporating data from subsequent screening cycles is required to assess temporal trends (transitory or persistent) and shed more light on the natural history of radiation-related effects on thyroid function.

## Supplemental Material

(606 KB) PDF
