# Supplementary material for: Measures of Thyroid Function among Belarusian Children and Adolescents Exposed to Iodine-131 from the Accident at the Chernobyl Nuclear Plant
Source: Environ Health Perspect. 2013 May 7;121(7):865–71. doi: 10.1289/ehp.1205783 (PMC3701991; doi:10.1289/ehp.1205783)
Supplement: (606 KB) PDF [file ehp.1205783.s001.pdf]

SUPPLEMENTAL MATERIAL

Measures of Thyroid Function among Belarusian Children and Adolescents Exposed to Iodine-131 from the Accident at the Chernobyl Nuclear Plant

Evgenia Ostroumova, Alexander Rozhko, Maureen Hatch, Kyoji Furukawa, Olga Polyanskaya, Robert J. McConnell, Eldar Nadyrov, Sergey Petrenko, George Romanov, Vasilina Yauseyenka, Vladimir Drozdovitch, Viktor Minenko, Alexander Prokopovich, Irina Savasteeva, Lydia B. Zablotska, Kiyohiko Mabuchi, and Alina V. Brenner

Table of Contents

Supplemental Material, Table S1. ORs and 95%CIs for selected risk factors associated with background prevalence of thyroid functional outcomes among individuals exposed to ^{131}I due to the Chernobyl accident **2-4**

Supplemental Material, Table S2. ORs of prevalence of thyroid functional outcomes by sex and age at examination among individuals exposed to ^{131}I due to the Chernobyl accident **5**

Supplemental Material, Table S1. ORs and 95%CIs for selected risk factors associated with background prevalence of thyroid functional outcomes among individuals exposed to ^{131}I due to the Chernobyl accident

Characteristics	Hypothyroidism Cases (%)	Hypothyroidism OR[a] (95% CI)	Hyperthyroidism Cases (%)	Hyperthyroidism OR[a] (95% CI)	Elevated ATPO Cases (%)	Elevated ATPO OR[a] (95% CI)	AIT Cases (%)	AIT OR[a] (95% CI)
Gender								
Male	137 (2.5)	1.00 (Referent)	49 (0.9)	1.00 (Referent)	198 (3.7)	1.00 (Referent)	12 (0.2)	1.00 (Referent)
Female	182 (3.3)	1.02 (0.79, 1.31)	88 (1.6)	1.56 (1.05, 2.34)	424 (7.8)	1.96 (1.62, 2.37)	75 (1.4)	5.13 (2.74, 10.41)
p-Value[b]		0.88		0.03		<0.001		< 0.001
Place of residency								
Gomel Oblast	216 (3.3)	1.52 (1.11, 2.10)	85 (1.3)	0.81 (0.53, 1.28)	367 (5.6)	0.99 (0.81, 1.22)	59 (0.9)	1.43 (0.83, 2.56)
Minsk Oblast & city	60 (1.9)	1.00 (Referent)	36 (1.2)	1.00 (Referent)	175 (5.6)	1.00 (Referent)	20 (0.6)	1.00 (Referent)
Mogilev & other oblasts	43 (3.6)	1.58 (1.02, 2.41)	16 (1.3)	1.00 (0.51, 1.86)	80 (6.6)	1.06 (0.79, 1.42)	8 (0.7)	0.91 (0.36, 2.12)
p-Value[b]		0.02		0.58		0.89		0.27
Residence								
Rural	161 (3.8)	1.23 (0.96, 1.57)	65 (1.5)	1.72 (1.18, 2.52)	214 (5.0)	0.80 (0.66, 0.96)	29 (0.7)	0.70 (0.42, 1.14)
Urban	158 (2.4)	1.00 (Referent)	72 (1.1)	1.00 (Referent)	408 (6.2)	1.00 (Referent)	58 (0.9)	1.00 (Referent)
p-Value[b]		0.10		0.005		0.02		0.15
Current smoking								
No	269 (3.6)	1.00 (Referent)	97 (1.3)	1.00 (Referent)	479 (6.4)	1.00 (Referent)	71 (0.9)	1.00 (Referent)
Yes	49 (1.5)	0.54 (0.38, 0.75)	38 (1.1)	0.95 (0.62, 1.45)	141 (4.3)	0.82 (0.66, 1.02)	15 (0.4)	0.86 (0.45, 1.55)
p-Value[b]		<0.001		0.83		0.07		0.64
Unknown	1 (2.6)	0.85 (0.05, 4.52)	2 (5.1)	2.29 (0.32, 10.00)	2 (5.1)	1.01 (0.15, 3.73)	1 (2.6)	9.03 (0.43, 63.84)
Vitamin consumption								
No	299 (3.0)	1.00 (Referent)	124 (1.2)	1.00 (Referent)	574 (5.8)	1.00 (Referent)	81 (0.8)	1.00 (Referent)
Yes	7 (2.3)	0.81 (0.34, 1.64)	1 (0.3)	0.27 (0.01, 1.23)	18 (5.9)	1.08 (0.63, 1.73)	2 (0.3)	0.83 (0.13, 2.80)
p-Value[b]		0.59		0.10		0.77		0.79
Unknown	13 (2.1)	0.68 (0.36, 1.15)	12 (1.9)	1.69 (0.86, 3.02)	30 (4.8)	0.78 (0.52, 1.12)	4 (0.6)	0.67 (0.20, 1.65)
Thyroid disease in relatives								
No	288 (2.9)	1.00 (Referent)	120 (1.2)	1.00 (Referent)	553 (5.7)	1.00 (Referent)	76 (0.8)	1.00 (Referent)
≥1 relative affected	22 (2.5)	0.72 (0.45, 1.11)	12 (1.3)	1.17 (0.60, 2.07)	60 (6.8)	1.13 (0.84, 1.48)	10 (1.1)	1.29 (0.57, 2.34)

Characteristics	Hypothyroidism Cases (%)	Hypothyroidism OR[a] (95% CI)	Hyperthyroidism Cases (%)	Hyperthyroidism OR[a] (95% CI)	Elevated ATPO Cases (%)	Elevated ATPO OR[a] (95% CI)	AIT Cases (%)	AIT OR[a] (95% CI)
p-Value[b]		0.14		0.62		0.40		0.61
Unknown	9 (5.3)	1.68 (0.75, 3.31)	5 (3.0)	1.92 (0.60, 4.81)	9 (5.3)	0.84 (0.39, 1.58)	1 (0.6)	0.37 (0.02, 2.09)
Year of exam								
1996 - 98	119 (4.0)	1.00 (Referent)	17 (0.6)	1.00 (Referent)	117 (3.9)	1.00 (Referent)	22 (0.7)	1.00 (Referent)
1999	57 (2.5)	0.70 (0.49, 0.98)	44 (1.9)	2.75 (1.56, 5.06)	195 (8.6)	2.30 (1.79, 2.96)	18 (0.8)	1.04 (0.54, 2.05)
2000 – 03	143 (2.6)	1.08 (0.79, 1.47)	76 (1.4)	1.72 (1.00, 3.11)	310 (5.5)	1.32 (1.04, 1.69)	47 (0.8)	1.34 (0.77, 2.37)
p-Value [b]		0.02		0.002		<0.001		0.57
Season of exam								
Dec – Feb	66 (2.7)	1.00 (Referent)	36 (1.5)	1.00 (Referent)	75 (3.1)	1.00 (Referent)	11 (0.4)	1.00 (Referent)
March – May	111 (2.5)	1.06 (0.77, 1.47)	59 (1.3)	0.75 (0.49, 1.17)	268 (6.0)	1.83 (1.41, 2.40)	38 (0.9)	1.71 (0.89, 3.59)
June – Aug	53 (3.2)	1.05 (0.71, 1.53)	15 (0.9)	0.48 (0.25, 0.89)	127 (7.8)	2.35 (1.75, 3.19)	16 (1.0)	1.22 (0.55, 2.78)
Sept – Nov	89 (3.8)	1.40 (1.01, 1.97)	27 (1.2)	0.73 (0.43, 1.23)	152 (6.5)	2.05 (1.54, 2.74)	22 (0.9)	2.03 (0.99, 4.44)
p-Value[b]		0.16		0.14		<0.001		0.18
ATPO level, U/mL								
≤ 60	257 (2.5)	1.00 (Referent)	111 (1.1)	1.00 (Referent)	0	n/a	6	n/a
> 60	62 (10.0)	3.58 (2.49, 5.08)	26 (4.2)	2.75 (1.60, 4.55)	622	n/a	81	n/a
p-Value[b]		<0.001		< 0.001				
ATG level, U/mL								
≤ 60	273	1.00 (Referent)	118	1.00 (Referent)	400	n/a	11	n/a
> 60	46	2.22 (1.47, 3.30)	19	1.51 (0.82, 2.68)	222	n/a	76	n/a
p-Value [b]		< 0.001		0.18				
Urinary iodine, µg/ L								
< 20	58 (4.8)	1.51 (1.06, 2.16)	9 (0.7)	0.59 (0.26, 1.21)	80 (6.6)	1.30 (0.97, 1.72)	15 (1.2)	1.51 (0.75, 2.96)
20 – 49	88 (3.1)	1.03 (0.75, 1.40)	18 (0.6)	0.57 (0.31, 1.01)	159 (5.6)	1.05 (0.83, 1.32)	14 (0.5)	0.64 (0.32, 1.24)
50 – 99	85 (2.8)	1.00 (Referent)	32 (1.0)	1.00 (Referent)	168 (5.5)	1.00 (Referent)	24 (0.8)	1.00 (Referent)
≥100	86 (2.4)	0.91 (0.67, 1.24)	75 (2.1)	2.10 (1.39, 3.25)	206 (5.7)	1.04 (0.84, 1.28)	33 (0.9)	1.18 (0.69, 2.05)
p-Value[b]		0.05		<0.001		0.35		0.11
Missing/ Unknown	2 (1.6)	0.54 (0.09, 1.78)	3 (2.4)	2.02 (0.47, 5.93)	9 (7.3)	0.99 (0.45, 1.95)	1 (0.8)	0.82 (0.04, 4.13)
Presence of goiter								
No	245 (2.6)	1.00 (Referent)	110 (1.2)	1.00 (Referent)	483 (5.2)	1.00 (Referent)	42 (0.45)	1.00 (Referent)
Yes	73 (4.8)	1.39 (1.04, 1.85)	26 (1.7)	1.64 (1.02, 2.57)	137 (8.9)	1.77 (1.43, 2.18)	45 (2.9)	7.50 (4.74, 11.90)

Characteristics	Hypothyroidism Cases (%)	Hypothyroidism OR[a] (95% CI)	Hyperthyroidism Cases (%)	Hyperthyroidism OR[a] (95% CI)	Elevated ATPO Cases (%)	Elevated ATPO OR[a] (95% CI)	AIT Cases (%)	AIT OR[a] (95% CI)
p-Value[b]		0.03		0.04		< 0.001		< 0.001
Unknown/ Missing	1 (3.8)	1.41 (0.08, 7.01)	1 (3.8)	2.46 (0.13, 12.86)	2 (7.7)	1.62 (0.26, 5.63)	0 (0.0)	0.0

Abbreviations: ATG, antibodies to thyroglobulin; ATPO, autoantibodies to thyroperoxidase; AIT, autoimmune thyroiditis; CI, confidence interval; OR, odds ratio; CI, confidence interval; n/a, not applicable.

[a]All analyses performed with adjustment for sex, sex and age at examination interaction, oblast of residency at examination, urban or rural residency, current smoking, vitamin consumption, family history of thyroid disease, year and season of examination, ATPO and ATG levels (except for elevated ATPO and AIT), urinary iodine levels, presence of goiter and ^{131}I thyroid dose based on a linear excess odds ratio model.

[b]*p*-Value for homogeneity.

Supplemental Material, Table S2. ORs of prevalence of thyroid functional outcomes by sex and age at examination among individuals exposed to ^{131}I due to the Chernobyl accident

Age at examination, years	Hypothyroidism Cases (%)	Hypothyroidism OR (95% CI)	Hyperthyroidism Cases (%)	Hyperthyroidism OR (95% CI)	Elevated ATPO Cases (%)	Elevated ATPO OR (95% CI)	AIT Cases (%)	AIT OR (95% CI)
Men								
11–14	45 (32.8)	1.00 (Referent)	1 (2.0)	1.00 (Referent)	21 (10.6)	1.00 (Referent)	1 (8.3)	1.00 (Referent)
15–19	56 (40.9)	0.65 (0.42, 1.00)	8 (16.3)	2.71 (0.49, 50.62)	53 (26.8)	0.96 (0.57, 1.65)	2 (16.7)	1.13 (0.11, 24.49)
20–24	16 (11.7)	0.22 (0.12, 0.40)	16 (32.7)	5.57 (1.11, 101.10)	54 (27.3)	1.13 (0.67, 1.96)	4 (33.3)	2.73 (0.39, 53.91)
25–33	20 (14.6)	0.24 (0.13, 0.43)	24 (49.0)	7.05 (1.45, 127.00)	70 (35.3)	1.36 (0.82, 2.35)	5 (41.7)	3.64 (0.57, 70.56)
p-Value for trend, df = 1	<0.001		0.002		0.07		0.09	
Women								
11–14	44 (24.2)	1.00 (Referent)	2 (2.3)	1.00 (Referent)	27 (6.4)	1.00 (Referent)	3 (4.0)	1.00 (Referent)
15–19	35 (19.2)	0.32 (0.20, 0.51)	18 (20.4)	2.73 (0.77, 17.32)	91 (21.5)	1.18 (0.76, 1.88)	13 (17.3)	2.05 (0.65-9.05)
20–24	49 (26.9)	0.44 (0.28, 0.70)	30 (34.1)	4.32 (1.28, 26.97)	126 (29.7)	1.65 (1.08, 2.61)	19 (25.3)	3.32 (1.10-14.38)
25–33	54 (29.7)	0.41 (0.26, 0.65)	38 (43.2)	4.89 (1.45, 30.46)	180 (42.4)	2.39 (1.57, 3.75)	40 (53.3)	7.48 (2.61-31.68)
p-Value for trend, df = 1	0.02		0.004		< 0.001		< 0.001	

Abbreviations: CI, confidence interval; OR, odds ratio; ATPO, autoantibodies to thyroperoxidase; AIT, autoimmune thyroiditis.

All analyses performed with adjustment for sex, oblast of residency at examination, urban or rural residency, current smoking, vitamin consumption, family history of thyroid disease, year and season of examination, ATPO and ATG levels (except for elevated ATPO and AIT), urinary iodine levels, presence of goiter and ^{131}I thyroid dose based on a linear excess odds ratio model.
